# Supplementary material for: Patterns of Intron Gain and Loss in Fungi
Source: PLoS Biol. 2004 Nov 30;2(12):e422. doi: 10.1371/journal.pbio.0020422 (PMC532390; doi:10.1371/journal.pbio.0020422)
Supplement: Table S1 — Also available at http://genes.mit.edu/NielsenEtAl/. (4.3 MB ZIP). [file pbio.0020422.st001.zip › NielsenEtAl/html/727.html]

AN5222.1.NCU03102.1.MG03680.1.FG00671.1


```
 CLUSTAL W (1.82) Multiple Sequence Alignments - Introns Inserted


Sequence 1: AN5222.1	160 aa
Sequence 2: FG00671.1	157 aa
Sequence 3: NCU03102.1	161 aa
Sequence 4: MG03680.1	161 aa
Alignment Length: 161 aa
Number Identitical Residues: 123 aa
Alignment Score (without introns) 5191


MG03680.1 	MATELTVQSERAFQKQPHI~FLNSKTKVKSARPGKGGRRYYKDVGLGFRTPKTAIEGQYI
NCU03102.1	MATELTVQSERAFQKQPHI1FLNSKTKVKSARPGKGGRRWYKDVGLGFRTPAAAIEGQYI
FG00671.1 	----MTVQSERAFQKQPHI~FQNSKTKTKSTRPGKGGRRWYKDVGLGFRTPKTAIEGSYI
AN5222.1  	MATELTVQSERAFQKQPHI~FLNPKTKAKSKKVGQT-RRWYKDVGLGFRTPKTAIEGSYI
          	 ::.:************** * *.***.** : *:  **:*********** :****.**

MG03680.1 	D1KKCPFTGLVSIRGRILNGTVISTKMHRTLVIRREYLHFIPKYARYEKRHKNLAAHVSP
NCU03102.1	D1KKCPFTGQVSIRGRILSGTVVSTKMHRTIIIRREYLHFIPKYNRYEKRHKNLAAHVSP
FG00671.1 	D1KKCPFTGLVSIRGRILTGTVVSTKMHRTIIIRREYLHFIQKYSRYEKRHKNVAAHVSP
AN5222.1  	D1KKCPFTGMVSIRGRILTGRVVSTKMHRTIIIRREYLHYVPKYNRYEKRHKNLAAHVSP
          	* ******* ********.* *:*******::*******:: ** ********:******

MG03680.1 	AFRVEEGDHVTVGQCRPLSKT0VRFNVLKVQPRSDKAVKSFAKF
NCU03102.1	AFRVEEGDKVTVGQCRPLSKT0VRFNVLRVLPRTGKSVKKFAKF
FG00671.1 	AFRVQEGDQVTVGQCRPLSKT0VRFNVLRVLPRTGKSVKKFSKF
AN5222.1  	AFRVEEGDWVTVGQCRPLSKT0VRFNVLRVLPRTGKAVKAFSKF
          	****:*** ************ ******:* **:.*:** *:**
```
